# Improving patient communication and understanding of medical reports through text simplification with ChatGPT: a comparative study

**DOI:** 10.1007/s10006-026-01643-4

**Published:** 2026-09-26

**Authors:** Daniel Stephan, Sophia Schumacher, Bilal Al-Nawas, Peer W. Kämmerer, Daniel G. E. Thiem

**Affiliations:** Department of Oral and Maxillofacial Surgery, Facial Plastic Surgery, University Medical Centre of the Johannes Gutenberg-University Mainz, Augustusplatz 2, 55131 Mainz, Germany

**Keywords:** Patient-centered communication, Natural language processing, Health literacy, Medical accessibility, Patient comprehension, Clinical communication

## Abstract

**Purpose:**

Medical documentation is essential for clinical communication but is often intended for professional audiences, limiting patient understanding. This linguistic complexity can reduce health literacy and hinder shared decision-making. Large language models offer new opportunities to simplify medical texts while maintaining factual accuracy, thereby improving accessibility and comprehension. This study aimed to evaluate whether ChatGPT can simplify medical reports while preserving clinical content and to assess whether these simplifications improve patient comprehension, readability, and perceived communication quality.

**Methods:**

Five document types were analysed, including MRI, CT, surgical, pathology reports, and discharge summaries. Each original physician-written document was simplified using a standardized ChatGPT prompt instructing full content preservation and patient-oriented phrasing. Ten simplifications per text were reviewed for completeness. Readability was measured using Flesch Reading Ease (FRE) and LIX indices. A total of 576 participants without medical knowledge evaluated either a simplified or original report version and completed a standardized questionnaire assessing clarity, structure, and applicability, followed by comprehension testing.

**Results:**

Simplified reports achieved significantly higher readability (FRE 48.4 ± 5.0 vs. 22.9 ± 5.2; *p* < 0.0001; LIX 48.3 ± 3.2 vs. 59.2 ± 2.5; *p* = 0.004). Across all document types, patients rated simplified texts significantly higher in clarity, structure, and usefulness (*p* < 0.001), with significantly improved comprehension accuracy (e.g., MRI 80.3% vs. 53.7%; *p* < 0.001). No loss of medical information was observed.

**Conclusions:**

ChatGPT appears to be capable of simplifying complex medical documents while preserving clinically relevant information, leading to improvements in both perceived readability and objective understanding under the conditions of this study. AI-driven text simplification thus represents a promising tool to enhance patient communication and health literacy.

**Supplementary Information:**

The online version contains supplementary material available at https://doi.org/10.1007/s10006-026-01643-4.

## Introduction

Medical documentation represents a core component of communication in clinical practice, ensuring that diagnostic findings, treatment plans, and follow-up recommendations are accurately conveyed among healthcare professionals [1]. Beyond its technical and legal functions, medical documentation also serves as a crucial instrument of patient communication. As healthcare increasingly emphasizes shared decision-making and patient participation, the accessibility of medical information has become a key determinant of high-quality patient-centered care [2, 3]. However, medical reports are often written in highly specialized language, characterized by abbreviations, anatomical terminology, and discipline-specific phrasing. For most patients, this complexity poses a major barrier to understanding their health status, treatment options, and recommendations.

Improving the readability and comprehensibility of medical communication is therefore essential to bridge the gap between professional documentation and patient understanding. Studies consistently show that greater linguistic accessibility correlates with improved patient satisfaction, adherence, and engagement in healthcare processes [4]. However, achieving a balance between clinical precision and lay accessibility remains difficult. Physicians must produce technically and legally accurate reports while facing growing administrative workloads that limit the opportunities for patient-focused explanations. Consequently, written communication often remains optimized for professional readers, reducing transparency and patient empowerment.

This challenge extends beyond medical reporting and is also evident in processes such as informed consent. Written informed consent, a fundamental element of autonomy-based medicine, aims to support shared decision-making between physicians and patients [5]. However, numerous studies have shown that patients frequently struggle to understand key aspects of consent forms, particularly regarding study design, risks, and procedures, despite their perceived comprehension [6, 7]. These results highlight a persistent barrier in medical communication: even when information is provided, inadequate linguistic accessibility can hinder true understanding and informed participation in healthcare decisions. Comparable comprehension deficits are also evident in everyday clinical care, particularly during patient discharge. Research consistently demonstrates that a substantial proportion of patients leave emergency departments without fully understanding their diagnosis, treatment, or follow-up instructions [8, 9]. Many patients fail to recall critical warning symptoms or adhere to discharge recommendations, regardless of whether the information is provided verbally or in written form. These findings emphasize that even routine clinical communication often fails to ensure adequate patient understanding, highlighting the need for clearer, more accessible information delivery.

Artificial intelligence (AI) offers a promising solution to this problem. Initially developed for data analysis and pattern recognition, AI is now increasingly applied to natural language processing tasks in medicine [10]. Large language models (LLMs), such as OpenAI’s Generative Pre-Trained Transformer (GPT), can autonomously generate coherent, contextually appropriate medical text [11–13]. Beyond text generation, AI systems are explored for text summarization and simplification, aiming to enhance the readability and accessibility of medical information while preserving clinical accuracy [14]. Among LLMs, ChatGPT has demonstrated strong capabilities across medical applications, including reasoning tasks, examination performance, and the generation of clinical text [15–19]. Prior studies have shown that ChatGPT can effectively rephrase complex medical language into patient-friendly terms without significant information loss [20, 21]. Nevertheless, such systems may produce linguistically fluent yet factually incorrect statements (“hallucinations”) [22, 23] and can perpetuate data-driven biases, potentially influencing equity in medical communication [24, 25]. Furthermore, as most models are trained primarily on English-language datasets, questions remain regarding their linguistic generalizability and cultural adaptability [26].

Despite these limitations, AI-driven text simplification offers a transformative opportunity to enhance medical communication, comprehension, and health literacy. A previous study from our group demonstrated that ChatGPT-simplified radiology reports significantly improved patient perceptions of clarity, empathy, and readability compared with original AI-generated texts [21]. Building on these findings, the present study extends this approach to five distinct medical document types: MRI and CT reports, surgical operation reports, pathology findings, and discharge summaries. To evaluate the effects of ChatGPT-based text simplification, the study combines subjective patient evaluations with objective comprehension testing. The study examines whether medical documents of different types can be simplified without loss of relevant clinical information and whether such simplifications lead to measurable improvements in patient understanding.

## Methods

The present study aimed to systematically evaluate whether large language models, specifically ChatGPT (OpenAI, San Francisco, CA), can improve patient communication by simplifying complex medical documents without loss of clinically relevant information. Both perceived clarity and actual comprehension of medical information were evaluated among patients to assess the impact of AI-based simplification. To address this objective, five representative medical document types were selected, each reflecting a distinct aspect of clinical communication: a magnetic resonance imaging (MRI) report, a computed tomography (CT) report, a surgical operating report, a pathology finding report, and a discharge summary. For each document type, an original physician-written version and a corresponding ChatGPT-simplified version were generated and subsequently evaluated by patients using standardized questionnaires. The evaluation included both subjective and objective outcome measures. Subjective perceptions of clarity, structure, tone, and information value were rated on a nine-item, five-point Likert questionnaire and objective comprehension was measured using multiple-choice questions derived from each report.

### Report simplification

Five anonymized medical documents were selected, each representing a common and clinically relevant type of written communication routinely provided to patients or accessible through electronic health records. These reports included a magnetic resonance imaging (MRI) report, a computed tomography (CT) report, a surgical operation report, a pathology report, and a medical discharge summary. Each original physician-written document was simplified using the large language model ChatGPT 4.0 (OpenAI, San Francisco, CA, USA) under the following parameters: temperature = 0.7 (controlling response variability), maximum token limit = 1500 (defining text length), frequency penalty = 0.0 (preventing repetitive phrasing), and presence penalty = 0.6 (encouraging lexical diversity). For each document, a context-specific prompt instructed ChatGPT to rephrase the text into accessible language suitable for laypersons with a reading level of approximately 11–12 years. The instructions explicitly required preservation of all clinically relevant information, exclusion of fabricated content, and maintenance of the original logical and structural coherence. To optimize the output, prompts were refined to account for the linguistic and stylistic characteristics of each document type resulting in minor report-specific adjustments to accommodate document-specific terminology. The final versions of all prompts are available in the supplementary materials. Each text was generated in a new ChatGPT web session using default settings with memory functions disabled, ensuring independence of all outputs. Report generation was conducted between October 19 and November 8, 2024.

### Content accuracy

Content accuracy of all ChatGPT-simplified texts was systematically assessed to verify the preservation of all clinically relevant information. To ensure reproducibility, each report was simplified ten times using the same standardized prompt. The outputs were reviewed for completeness, factual accuracy, and adherence to the prompt instructions. A checklist-based evaluation framework was developed for this purpose: each original document was systematically categorized according to its medical content, including findings, diagnoses, procedures, and results. All ten simplified versions were then compared against checklist derived from the original report to identify missing information. One representative version per document type, confirmed to contain all relevant content, was selected for subsequent evaluation.

### Readability indices

The readability and linguistic complexity of all texts were evaluated using the Flesch Reading Ease (FRE) score [27] and the LIX readability index [28, 29], as described in detail in our previous publications [13, 21]. Both metrics assess text comprehensibility based on structural and lexical characteristics, such as sentence length and word complexity. Higher FRE scores indicate more readable texts, whereas higher LIX values reflect greater linguistic complexity. Both indices are well-established and widely validated in medical and non-medical contexts, providing a reliable quantitative measure for comparing readability across text versions [30, 31].

### Study setting and participants

The study was conducted at the Department of Oral and Maxillofacial Surgery, Facial Plastic Surgery, University Medical Center Mainz, Germany. Patients were randomly selected and invited to participate. Participants were assigned to study groups using a computer-based randomization procedure implemented within the online survey platform (https://www.soscisurvey.de; SoSci Survey GmbH, Munich, Germany). The platform utilizes a random generator to assign participants to experimental groups, ensuring an automated and approximately balanced distribution across conditions. Upon accessing the survey, participants were automatically allocated to one of the study groups without any manual intervention. Due to the fully automated and anonymous assignment process, no influence on group allocation by investigators was possible. After providing informed consent, each participant was presented with one randomly assigned medical document and the corresponding questionnaire. To avoid bias from repeated exposure, participants were allowed to complete the survey only once and were therefore assigned to a single document type. Individuals with prior medical education or professional experience in healthcare, as well as participants under 18 years of age, were excluded. No additional exclusion criteria were applied to include a broad and representative sample of the general patient population typically encountered in clinical practice. Both participants and observer were blinded to group allocation, and all document versions were fully anonymized. Data collection was performed anonymously via a secure online survey platform between January 15 and June 2, 2025, without recording any personal data. All participants were informed about the study objectives and their right to withdraw participation at any time without disadvantage.

### Experimental design

Participants were randomly assigned to one of ten study groups, each receiving one medical document (MRI report, CT report, surgical report, pathology report, or discharge summary) in either its original or simplified version, along with the corresponding questionnaire. Each participant evaluated only one document type to prevent bias from repeated exposure. All responses were collected and analyzed anonymously. The questionnaire comprised two sections. The first section included nine items grouped into three thematic categories: comprehensibility (items 1–3), structure & information (items 4–6), and application & interaction (items 7–9), and were rated on a five-point Likert scale, ranging from 1 (“do not agree at all”) to 5 (“fully agree”), with intermediate options: “rather do not agree” (2), “neither agree nor disagree” (3) and “rather agree” (4). These items assessed participants’ subjective perceptions of clarity, structure, and overall usefulness of the text. The second section consisted of five multiple-choice comprehension questions designed to objectively evaluate participants’ understanding of the medical content. The same comprehension questions were used for both the original and simplified versions of each document type to ensure direct comparability. The full set of both rating and comprehension questions is provided in the supplementary materials. No time restrictions were imposed during the survey, allowing participants to read and respond at their own pace to ensure the reflection of genuine comprehension rather than the effects of time pressure or cognitive fatigue.

### Ethical approval

This study was conducted in full accordance with established ethical standards. All participants provided informed consent prior to participation, following a detailed explanation of the study’s purpose and procedures. Confidentiality and data protection were maintained at every stage to safeguard participant privacy and well-being. The Ethics Committee of the State Medical Association of Rhineland-Palatinate (Mainz, Germany) reviewed the study protocol and confirmed that formal ethical approval was not required, as the project represented a quality assurance measure and all data were collected and analyzed anonymously (Application Number: 2024-17526).

### Power analysis and sample size

The sample size was determined a priori based on the findings of our prior study, which demonstrated that simplified radiology reports were rated significantly higher regarding comprehensibility, usefulness, and perceived communication quality compared to the original versions with large effect sizes (Cohen’s d > 0.8; p < 0.001) [21]. Based on these results and assuming a similar effect size, a minimum of n = 26 participants per group (original vs. simplified version) is required to achieve a power of 80% and maintain a significance level of 5%. Considering the five different document types, this corresponds to 52 participants per document type and a total of 260 participants.

### Statistical analysis

Statistical analyses were conducted using the following software packages: GraphPad Prism 10.0 (GraphPad Software, LLC, Boston, USA), Excel 16.76 (Microsoft Corporation, Redmond, USA), and SPSS Statistics 29 (IBM Deutschland GmbH, Böblingen, Germany). G*Power 3.1 (Heinrich-Heine-University Düsseldorf, Düsseldorf, Germany) was used to calculate the required sample size. ChatGPT 5.0 was used for language refinement and proofreading. Differences in patient-rated comprehensibility, structure, and informational quality across text types (original vs. ChatGPT-simplified) were analyzed using nonparametric Mann–Whitney U tests. Data are presented as medians and interquartile ranges (IQR), and visualized using box-and-whisker plots. Response data from the multiple-choice comprehension questions were analyzed using two-sided Fisher’s exact test. Group-wise comparisons of correct response rates were performed for each text type separately. Proportional values are reported as percentages of correct responses per total answers, with statistical significance set at p < 0.05. Readability scores were calculated for each text version using established formulas [13, 21]. Comparative analysis of readability metrics between original and simplified text pairs was conducted using paired two-tailed t-tests.

### Note on language and translation

The entire study was conducted in German. All study materials, including prompts and questionnaire items, were originally developed and administered in German. For presentation purposes within this publication, these elements were translated into English, as this could be done without loss of meaning. However, the original and simplified medical texts themselves were not translated, since the translation into English would have altered linguistic nuances and readability characteristics that were central to the investigation. Retaining the original German versions therefore ensures the validity of the linguistic analyses and preserves the authenticity of the patient experience.

## Results

Content accuracy, readability, and clarity of original and ChatGPT-simplified medical reports were evaluated across five document types: MRI reports, CT reports, surgical reports, pathology reports, and discharge summaries. In addition, patient responses to comprehension questions were analysed to evaluate the effectiveness of text simplification in enhancing understanding and engagement with medical information. The findings indicate that the simplified versions substantially enhanced readability, clarity, and perceived accessibility, with patients achieving significantly higher comprehension scores for the simplified texts.

### Content accuracy

For all text types, each of the ten repetitively simplified versions retained the full medical content of the corresponding original report. The simplification process, performed repeatedly for validation, did not result in any loss of relevant information.

### Simplified text versions demonstrated improved readability

**Fig. 1:**
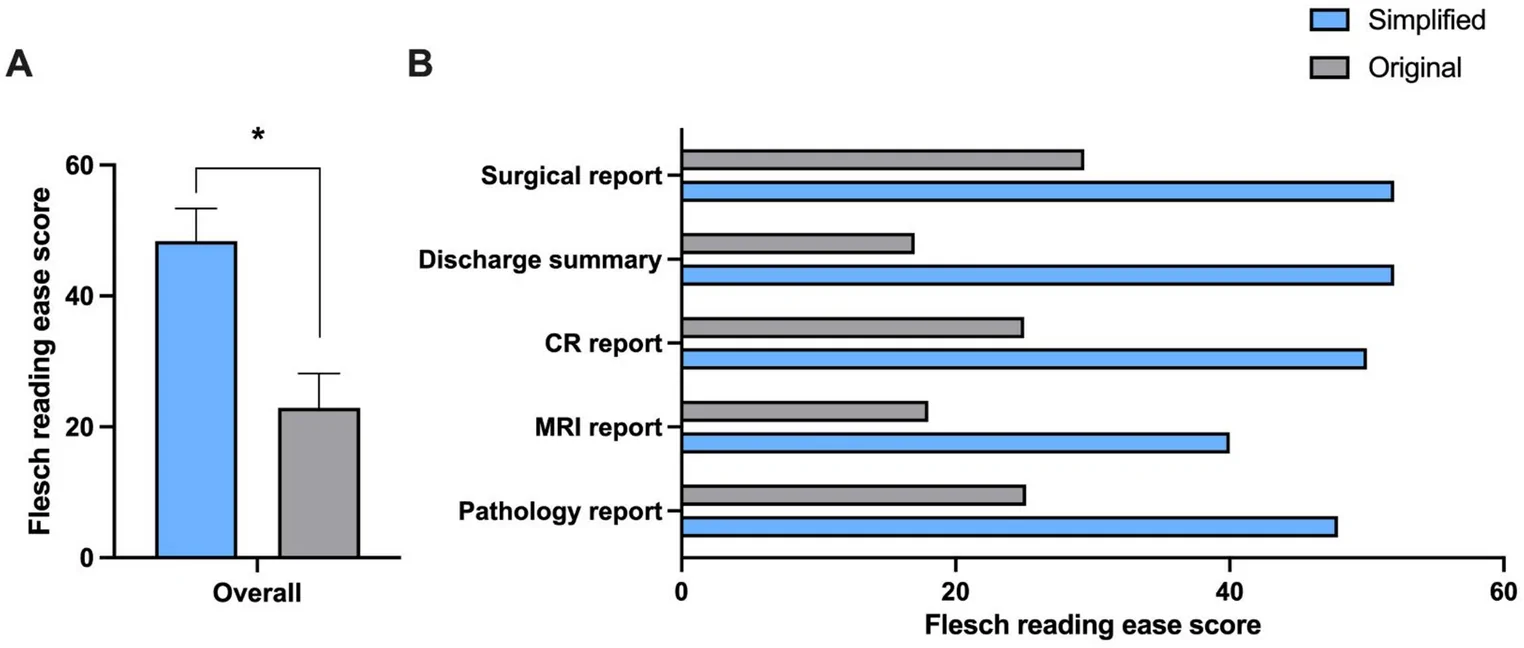
Metric evaluation of readability of original and AI-simplified reports medical reports assessed with the Flesch Reading Ease score presented overall (**A**) and separately for each text type (**B**). Data represent mean +/- SEM. Sample size: *n* = 5 (**A**); *n* = 1 (**B**); *p < 0.05

Significant differences in readability were observed with significantly higher Flesch Reading Ease scores for the simplified versions compared to the original medical reports (Fig. 1A: 48.4 ± 5.0 vs. 22.9 ± 5.2; p < 0.0001; t = 10.45, df = 4). When evaluated for individual text types, simplified reports consistently showed improved readability, reflected by higher Flesch Reading Ease scores compared to the original versions for all five document categories (Fig. 1B). As each comparison represents a single text pair, no statistical testing was applied for these values.

**Fig. 2:**
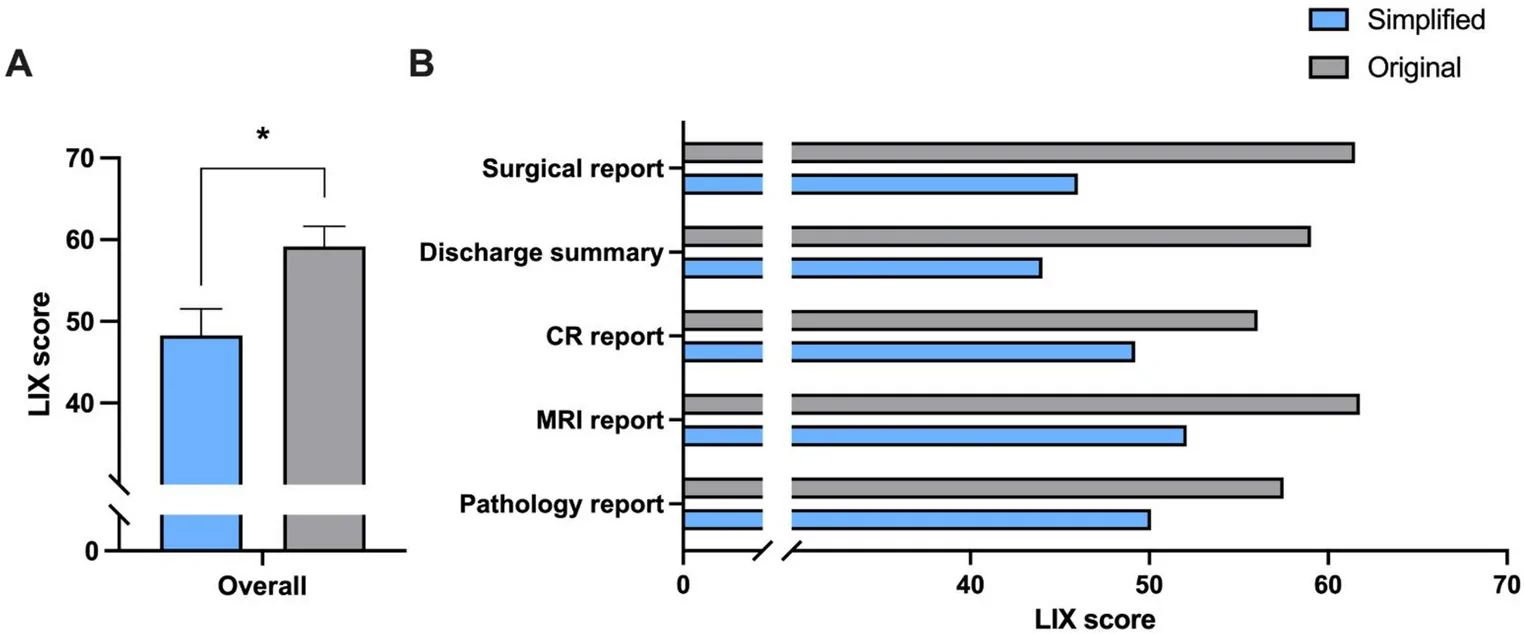
Metric evaluation of readability of original and AI-simplified reports medical reports assessed with the LIX score presented overall (**A**) and separately for each text type (**B**). Data represent mean +/- SEM. Sample size: *n* = 5 (**A**); *n* = 1 (**B**); *p < 0.05

As depicted in Fig. 2, similar results to those shown in Fig. 1 could be observed in the LIX scores. LIX scores for simplified texts were significantly lower compared to original reports both overall (Fig. 2A: 48.3 ± 3.2 vs. 59.2 ± 2.5; p = 0.004; t = 5.90, df = 4) and across individual text types (Fig. 2B).

### Descriptive statistics of participant ratings and comprehension accuracy

A total of 576 participants completed the survey, with 289 evaluating the simplified and 287 the original text versions, as summarized in Table 1. Gender distribution was balanced between groups (simplified: 128 male, 161 female; original: 115 male, 172 female). Overall, more rating questionnaires than comprehension questionnaires were completed, indicating a higher dropout rate for comprehension tasks. However, dropout rates did not differ between simplified and original texts. Across all text types, 473 rating responses (simplified: n = 241; original: n = 232) and 439 comprehension responses (simplified: n = 229; original: n = 210) were collected. The overall number of completed questionnaires was therefore comparable between both text versions, with slightly fewer participants completing the comprehension section than the rating section across all document types.

**Table 1:** Descriptive statistics of participant responses for original and simplified medical reports. Data represent the number of participants (male, female, total) and the number and percentage of completed questionnaires for rating (Rating) and comprehension (Comp.) tasks across all text types (MRI report, CT report, surgical report, pathology report, and discharge summary). “Rating” refers to subjective evaluations of text comprehensibility, structure and information and application and interaction, while comprehension refers to completed comprehension-questionnaire responses assessing understanding of the report content

Text Type		M	F	Total	Rating (n)	Rating (%)	Comp. (n)	Comp. (%)
Simplified	MRI	25	33	58	51	87,9	47	81,0
	CT	23	36	59	42	71,2	40	67,8
	Surgical	22	36	58	48	82,8	46	79,3
	Pathology	29	28	57	51	89,5	47	82,5
	Discharge	29	28	57	49	86,0	49	86,0
	Total	128	161	289	241	83,4	229	79,2
Original	MRI	27	31	58	45	77,6	41	70,7
	CT	27	29	56	51	91,1	48	85,7
	Surgical	20	38	58	47	81,0	40	69,0
	Pathology	20	38	58	44	75,9	40	69,0
	Discharge	21	36	57	45	78,9	41	71,9
	Total	115	172	287	232	80,8	210	73,2
	Total	243	333	576	473	439	82,1	76,2

### Simplified medical reports received consistently higher patient ratings

**Fig. 3:**
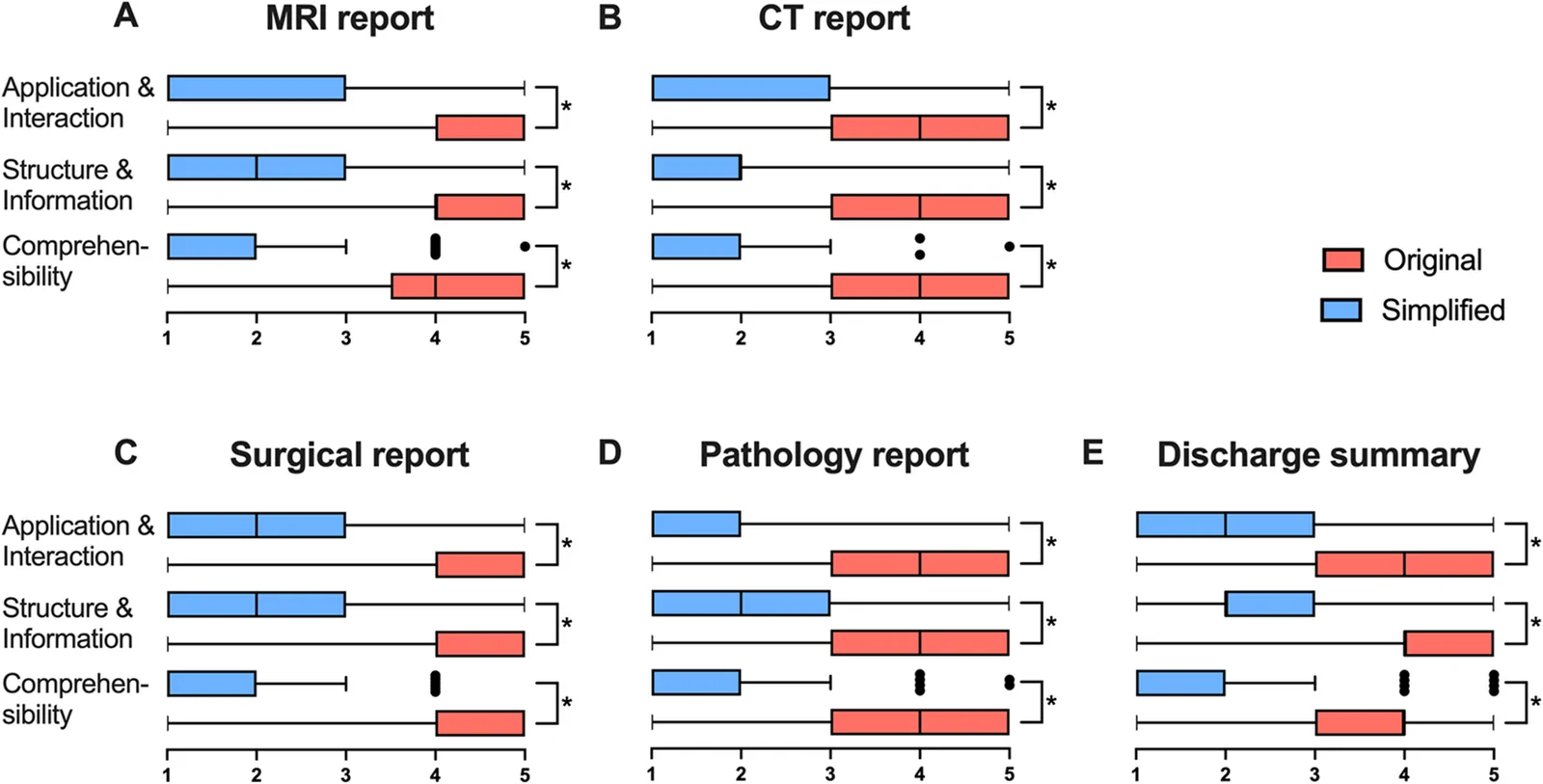
Patient ratings of original and simplified medical reports across all five document types. Patient evaluations were obtained for five medical document types: MRI report (**A**), CT report (**B**), surgical report (**C**), pathology report (**D**), and discharge summary (**E**) and assessed across three predefined categories: comprehensibility, structure & information, and application & interaction. Ratings were collected on a 5-point Likert scale ranging from 1 (“do not agree at all”) to 5 (“fully agree”). Data are presented as box-and-whisker plots showing medians and interquartile ranges. Statistical analysis was conducted using the Mann–Whitney U test. Sample sizes for each comparison are detailed in the results section; *p < 0.05

Across all five medical document types, the simplified versions received consistently higher patient ratings compared to the original reports in all evaluated categories (comprehensibility, structure and information, application and interaction) as assessed on a 5-point Likert scale. Statistical analysis using Mann–Whitney U test confirmed significant differences. As presented in Fig. 3A, for MRI reports, simplified texts received significantly higher ratings for comprehensibility (U = 1932, p < 0.001; n = 153 / 135), structure & information (U = 2121, p < 0.001; n = 153 / 135), and application & interaction (U = 2133, p < 0.001; n = 153 / 135). This result was consistent for CT reports (Fig. 3B: comprehensibility: U = 1842, p < 0.001; n = 126 / 153; structure & information: U = 3562, p < 0.001; n = 126 / 153; application & interaction: U = 2498, p < 0.001; 132 / 149), as well as for surgical reports (Fig. 3C: comprehensibility: U = 1186, p < 0.001; n = 144 / 141; structure & information: U = 2610, p < 0.001; n = 144 / 141 ; application & interaction: U = 1618, p < 0.001; n = 142 / 141). Similarly, pathology reports and discharge summaries demonstrated the same trend, with simplified versions receiving significantly higher evaluations across all dimensions as depicted in Fig. 3D (comprehensibility: U = 2184, p < 0.001; n = 147 / 134; structure & information: U = 2716, p < 0.001; n = 147 / 132; application & interaction: U = 3116, p < 0.001; n = 147 / 132) and Fig. 3E (comprehensibility: U = 2018, p < 0.001; n = 138 / 115; structure & information: U = 2889, p < 0.001; n = 138 / 114; application & interaction: U = 2889, p < 0.001; n = 138 / 114).

### Simplified text versions received higher ratings across all questionnaire items

**Fig. 4:**
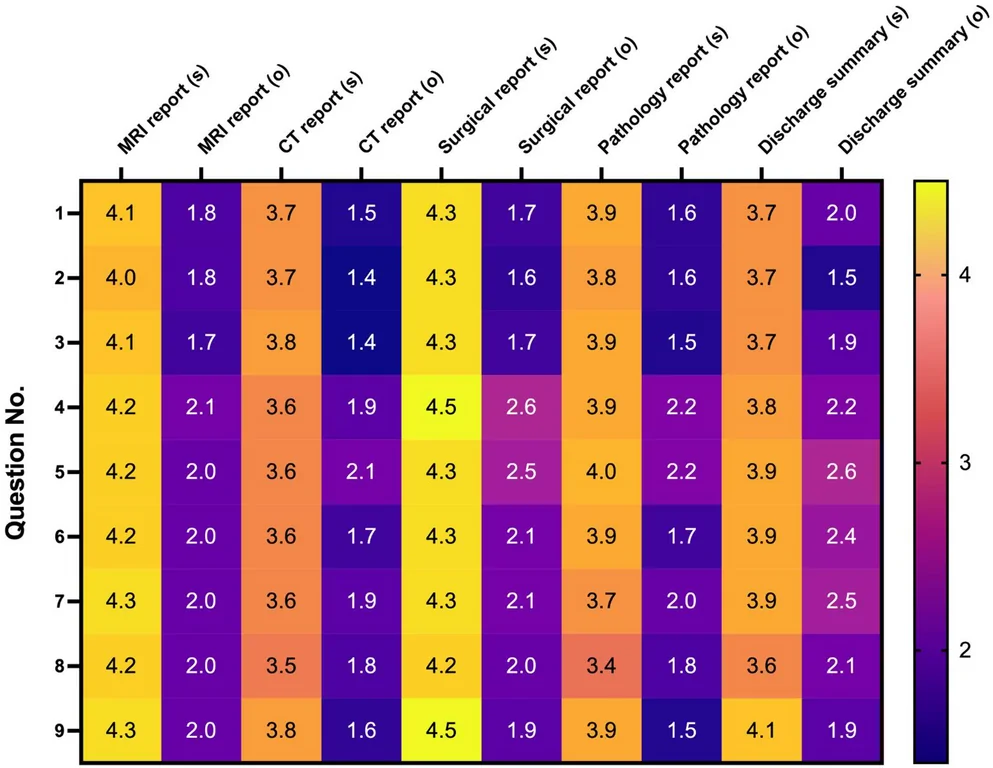
Median patient ratings across all assessed items for simplified (s) and original (o) medical reports, visualized as a heatmap. Ratings were obtained for nine assessment items across five document types (MRI report, CT report, surgical report, pathology report, and discharge summary) using a 5-point Likert scale (1 = “do not agree at all”, 5 = “fully agree”). Mann–Whitney U tests were used for statistical comparison between versions. Full statistical results are reported in the Supplementary Files

Figure 4 illustrates the distribution of patient ratings across all nine questionnaire items for each document type. Simplified versions consistently achieved higher median ratings across nearly all assessed items, confirming the improvement of perceived clarity, structure, and overall usability. The trend was observed across all document types, with no decrease in scores in any category. Detailed statistical results for individual items and text types are provided in table 2 in the supplementary files.

### Simplified reports significantly improved patient comprehension

**Fig. 5:**
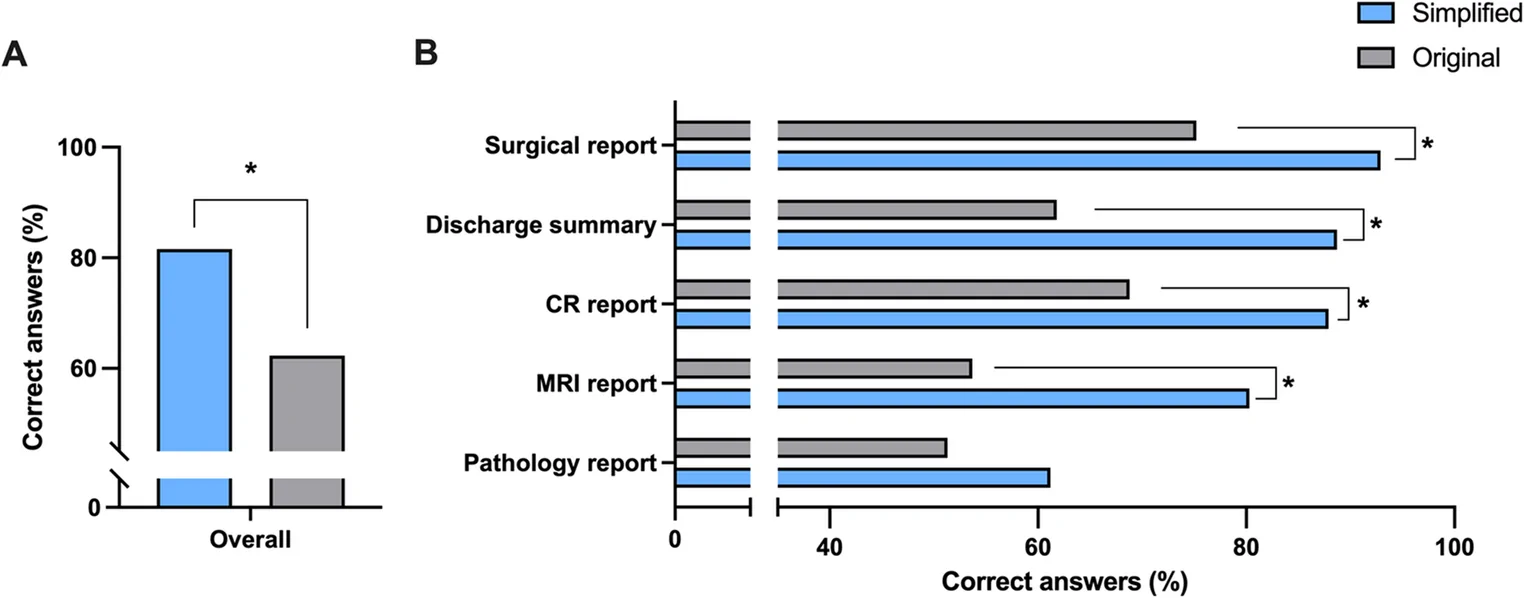
Patient comprehension performance for simplified and original medical reports with overall (**A**) percentage of correctly answered comprehension questions and individual (**B**) percentage of correct answers across five medical document types (MRI reports, CT reports, surgical reports, pathology reports, and discharge summaries). Statistical comparisons were conducted using unpaired t-tests (**A**) and Fisher’s exact tests (**B**). Sample sizes for each comparison are detailed in the results section; *p < 0.05

Simplified versions of all medical document types resulted in clearly enhanced comprehension performance compared to the original reports. Overall, participants answered a significantly higher proportion of comprehension questions correctly after reading the simplified texts (Fig. 5A: 82.2 ± 12.6 % vs. 62.2 ± 10.1 %; p = 0.023; t = 2.782, df = 8; n = 898 / 801). Consistent improvements were observed across individual document types as revealed by Fisher’s exact test (Fig. 5B). Participants demonstrated significantly higher comprehension accuracy for simplified surgical reports (92.9 % vs. 75.2 %; p < 0.001; n = 184 / 157), discharge summaries (88.7 % vs. 61.8 %; p < 0.001; n = 196 / 158), CT reports (87.9 % vs. 68.8 %; p < 0.001; n = 157 / 186), and MRI reports (80.3 % vs. 53.7 %; p < 0.001; n = 188 / 164). For pathology reports, comprehension rates were higher for the simplified version (61.2 % vs. 51.3 %; n = 168 / 136), although this difference was not significant (p = 0.0669).

**Fig. 6:**
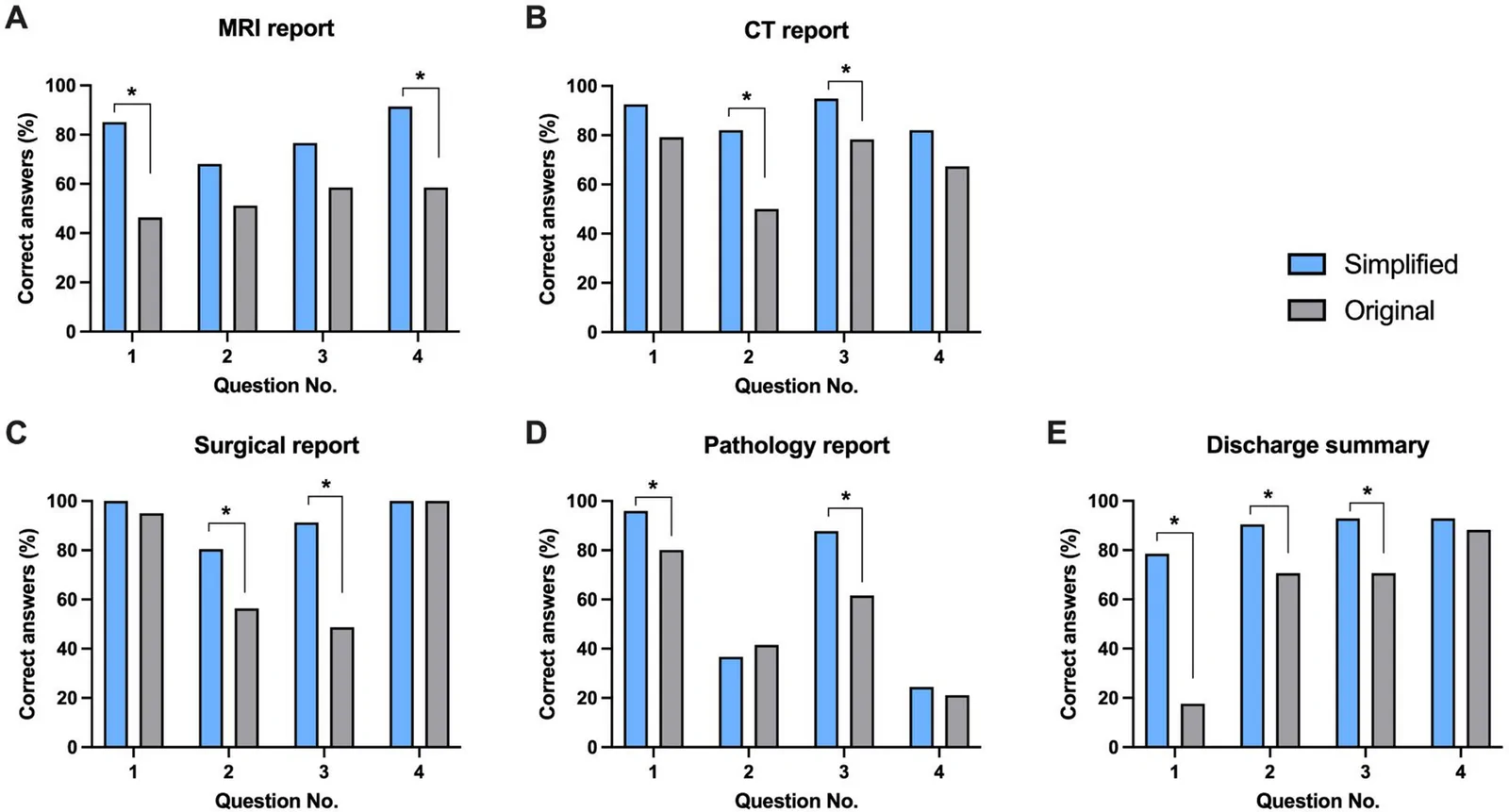
Percentage of correctly answered comprehension questions for simplified and original medical reports, with four comprehension questions per text type. Data are presented as percentages of correct answers. Statistical comparisons were performed using Fisher’s exact test. Sample sizes for each comparison are detailed in the results section; *p < 0.05

Across all five medical document types, simplified report versions consistently led to higher comprehension accuracy across individual questions. Participants who read the simplified MRI report (Fig. 6A) answered a significantly greater proportion of questions correctly, particularly for question 1 (p < 0.001) and question 4 (p < 0.001). Similarly, for CT reports (Fig. 6B), comprehension performance improved significantly for question 2 (p = 0.003) and question 3 (p = 0.032). In the surgical report group (Fig. 6C), simplified versions also yielded higher accuracy for question 2 (p = 0.02) and question 3 (p < 0.001). For pathology reports (Fig. 6D), statistically significant differences were observed for question 1 (p = 0.04) and question 3 (p = 0.005). Finally, patients demonstrated markedly better comprehension for the simplified discharge summaries (Fig. 6E), answering questions 1 (p < 0.001), 2 (p = 0.037), and 3 (p = 0.014) correctly more often compared to reading the original versions. 

## Discussion

The integration of artificial intelligence into clinical routine is most evident in medical documentation due to its potential to improve both the efficiency of healthcare workflows and the accessibility of medical communication. Based on previous results of our group, demonstrating that ChatGPT can enhance patient perceptions of clarity, empathy, and readability of radiology reports [21], this study extended this approach to five distinct types of medical documents: MRI and CT reports, surgical operation reports, pathology reports, and discharge summaries. Simplification by ChatGPT resulted in a significant overall improvement in text readability, as indicated by higher Flesch Reading Ease scores and lower LIX indices. Across all text types, AI-simplified versions were rated significantly higher by patients regarding comprehensibility, structure and information, and application and interaction. Importantly, these improvements in perceived clarity were accompanied by measurable gains in objective understanding. Participants answered a significantly greater proportion of comprehension questions correctly after reading the simplified texts compared to the original reports. Repeated analyses confirmed that no medical information was lost during the simplification process, suggesting that ChatGPT was able to preserve factual content across multiple iterations under experimental conditions.

These results expand the current understanding of AI-assisted communication in medicine. While previous research has primarily focused on the generation of technically accurate medical reports for professional use, this study underscores the equally critical importance of linguistic accessibility for patients. Most medical documentation is traditionally written for clinicians and contains highly specialized terminology, abbreviations, and implicit assumptions making it challenging for non-experts to understand. These linguistic barriers contribute directly to limited health literacy, a well-established determinant of patient engagement and health outcomes [2, 3, 32]. Lower health literacy often leads to difficulties in understanding diagnoses, interpreting test results, or following treatment instructions, which in turn is associated with poorer adherence, reduced participation in shared decision-making, and worse long-term outcomes. Prior studies have further repeatedly demonstrated that patient-facing materials in most medical specialties exceed recommended readability thresholds [33, 34]. For example, educational resources in oncology and cardiology are commonly written at a university level, despite public health authorities recommending a sixth-grade reading level for patient materials [34]. This mismatch illustrates the persistent disparity between professional communication standards and patients’ comprehension capacities. The present study provides evidence that AI-based text simplification can bridge this gap, achieving a balance between medical accuracy and linguistic accessibility. By maintaining content integrity while improving clarity, ChatGPT may offer a scalable approach to address communication inequities across medical disciplines.

This capability reflects the progress in large language models (LLMs), which have demonstrated an increasing ability to adapt language complexity to different audiences while preserving contextual meaning. LLMs such as GPT-4 and GPT-5 thus offer new opportunities to improve medical communication by dynamically adjusting linguistic complexity and summarizing domain-specific information without compromising essential content [14, 35]. The present results confirm that LLMs can be effectively applied not only for summarization or translation but also for the targeted simplification of patient-directed medical documents. Building on previous findings that ChatGPT-simplified radiology reports were perceived as more empathetic and respectful, reflecting improvements in tone and interpersonal communication quality, the current study focused on structural and informational aspects of textual clarity. Across all document types, simplified versions were rated significantly higher in comprehensibility, structure and information, and application and interaction, indicating that linguistic simplification enhances both perceived and functional accessibility. Importantly, these improvements extended beyond subjective impressions, represented by significantly higher scores in objective comprehension testing, confirming that simplification facilitates not only easier reading but genuine understanding of medical content. These findings therefore suggest that accessibility in medical communication depends on more than the replacement of complex terminology. Structural organization, logical coherence, and linguistic clarity play equally important roles enabling patients to process and retain medical information. The consistent ability of ChatGPT to generate logically structured, linguistically accessible, and content-preserving text supports its potential as a complementary tool for clinicians. By automating the simplification of reports and discharge letters, LLMs may help reduce administrative burden and allow healthcare professionals to dedicate more time to direct, personal communication with patients. Moreover, they enable the creation of medical documents that serve multiple audiences simultaneously: a single report can meet professional communication standards while being automatically reformulated into a patient-accessible version that retains all clinical content. This capacity to address both medical and non-medical readers without increasing the clinician’s workload represents a major practical advantage, promoting efficiency, transparency, and inclusivity in clinical communication.

An important methodological aspect of this study is the combination of subjective and objective measures of understanding. Whereas many previous investigations have relied primarily on readability metrics or expert assessments, the inclusion of comprehension testing in this study allows for a more direct evaluation of the cognitive effects of text simplification. Participants’ improved performance on comprehension questions indicates that simplification facilitated not only reading fluency but also information processing and recall. Notably, the magnitude of improvement was consistent across several document types. Gains were most pronounced for radiology and surgical reports as well as discharge summaries, representing document types known for their high technical density and critical importance for patient guidance. Although comprehension improved for pathology reports, this difference did not reach statistical significance, which may be explained by the particularly high linguistic and conceptual complexity of pathology reports, including highly specialized terminology and abstract diagnostic classifications that remain challenging to fully translate into lay language. Overall, these findings suggest that AI-assisted simplification appears to be robust across medical contexts, capable of improving understanding even when faced with highly specialized or complex material.

Despite the encouraging results, several limitations should be considered. First, the study was conducted in German within a single linguistic and cultural setting, which may limit the generalizability of the findings to other languages and healthcare systems. Linguistic structure, medical terminology, and readability metrics vary across languages and may influence both simplification performance and comprehension outcomes. In particular, languages differ in syntactic complexity, word formation, and the transparency of medical terminology, which may affect how effectively complex content can be transformed into patient-friendly language. It is therefore conceivable that the magnitude of improvement observed in this study may vary across languages, although the underlying principle of AI-based simplification is likely to remain applicable. Future research should investigate AI-based text simplification in multilingual settings to assess its generalizability across different linguistic and cultural contexts. In addition, information on participants’ educational background was not collected due to the fully anonymous study design. As educational level is closely associated with health literacy, future studies should incorporate this variable to allow a more detailed interpretation of comprehension outcomes. Furthermore, participants were recruited from a single clinical department, which may introduce selection bias and limit the representativeness of the study population. Individuals presenting to an oral and maxillofacial surgery setting may differ from the general population in terms of age distribution, educational background, and familiarity with medical or dental terminology. As no detailed demographic data beyond gender or baseline measures of health literacy were collected, the extent to which these factors may have influenced comprehension outcomes cannot be fully assessed. Future studies should therefore include more diverse and representative populations, as well as standardized assessments of health literacy, to further validate the generalizability of these findings. Second, although content completeness was systematically verified, detailed semantic validation by independent medical experts was not performed for every output. Additional expert review would further strengthen conclusions regarding medical accuracy. Moreover, while the multiple-choice comprehension questions provided an objective measure of understanding, they may not fully capture deeper conceptual understanding, inferential reasoning, or long-term information retention. Complementary qualitative approaches, such as open-ended questioning or patient interviews, could offer insights into how AI-simplified texts influence comprehension and trust and should be considered in further research. Third, this study focused exclusively on written medical communication. The potential of AI-assisted simplification to improve comprehension in other modalities (e.g. spoken explanations, audiovisual materials, interactive digital interfaces) remains to be explored. Additionally, the investigation was limited to single-timepoint evaluation, hence longitudinal studies will be required to assess whether improved comprehension translates into lasting knowledge retention, behavioural changes, or enhanced patient–physician interactions over time.

Finally, while ChatGPT performed reliably under the controlled experimental conditions of this study, its outputs may still vary depending on model updates, prompt formulation, or contextual framing, leaving critical considerations regarding the broader use of LLMs in clinical communication. The finding that no medical information was lost or fabricated across ten repeated simplification cycles provides reassurance of the model’s internal consistency and factual stability. This robustness addresses one of the key concerns associated with LLMs, namely the risk of “hallucinations” or fabricated content [22], and suggests that, under defined and supervised conditions, AI can operate dependably in bounded linguistic transformation tasks such as simplification. In this study, standardized prompts explicitly instructed ChatGPT to simplify medical texts without omitting or altering clinical information, resulting in consistently accurate and content-preserving outputs. These results highlight the importance of precise, context-aware prompt engineering as a central determinant of model reliability [36, 37]. Developing standardized prompt frameworks and validation protocols will therefore be essential to ensure reproducibility, transparency, and trustworthiness as AI tools become more integrated into healthcare documentation and communication workflows. However, this integration entails important ethical and professional responsibilities. Questions of authorship, accountability, and data protection must be clearly addressed before AI-generated content can be safely implemented in clinical practice. Physicians remain ultimately responsible for the accuracy and appropriateness of the information provided to patients, underscoring the need for institutional oversight and transparent review mechanisms. AI tools such as ChatGPT should thus be regarded as complementary aids rather than replacements for human communication. Overreliance on automated text generation risks diminishing trust, empathy and dialogue potentially impairing the sensible physician–patient relationship.

Overall, ChatGPT appears to be capable of simplifying various medical documents while preserving clinically relevant information under the controlled conditions of this study, leading to measurable improvements in both readability and patient comprehension. These findings highlight the potential of AI-assisted text simplification to make complex medical information more accessible and therefore strengthen patient understanding as foundation of informed decision-making. Comprehensible documentation may further reduce patient anxiety, support adherence to follow-up recommendations, and facilitate clearer communication during consultations. As healthcare systems continue to expand digital access to medical records, the integration of AI tools such as ChatGPT into documentation workflows could enable the automated generation of patient-friendly report versions with minimal additional effort for clinicians. When applied responsibly and under professional supervision, AI-driven simplification can contribute to more transparent, equitable, and patient-centered healthcare communication.

## Supplementary Information

Below is the link to the electronic supplementary material.

Supplementary Material 1 (DOCX 49.4 KB)

## Data Availability

No datasets were generated or analysed during the current study.

## Declarations

### Annotation

This study was conducted as part of Sophia Schumacher’s doctoral thesis.
