# Supplementary material for: Improving patient communication and understanding of medical reports through text simplification with ChatGPT: a comparative study
Source: Oral Maxillofac Surg. 2026 Sep 26;30(1):154. doi: 10.1007/s10006-026-01643-4 (PMC13614978; doi:10.1007/s10006-026-01643-4)
Supplement: Supplementary file 1 [file 10006_2026_1643_MOESM1_ESM.docx]

Supplementary Material

Prompts:
MRI report:
Rewrite the following medical report in simple and understandable language, without omitting important information or introducing any misinformation. Please follow these instructions when simplifying the text: Explain all medical terms and abbreviations in plain language. Briefly describe procedures or pathological terms in simple language (preferably in parentheses after the technical term). Retain all essential information, including findings, diagnoses, operations, reports, clinical course, and outcomes. Ensure that all treatment steps and medical procedures are represented accurately and completely. Do not shorten or omit any information relevant to the medical treatment. Do not invent or add any content that is not explicitly mentioned in the original report. Write the output as a continuous narrative text (no bullet points). 

CT report:
Rewrite the following medical report in simple and understandable language, approximately at the reading level of an 11–12-year-old, without omitting important information or introducing any misinformation. Please follow these instructions when simplifying the text: Explain all medical terms and abbreviations in plain language. Briefly describe procedures or pathological terms in simple language, preferably in parentheses after the technical term. Retain all essential information, including findings, diagnoses, operations, reports, clinical course, and outcomes. Ensure that all treatment steps and medical procedures are reproduced accurately and completely. Do not shorten the content or omit any details relevant to medical care. Do not add or invent any information that is not explicitly mentioned in the original report. Write the output as a continuous narrative text (no bullet points). Preserve the structure and sectioning of the original report.

Surgical report:
Rewrite the following medical report in simple and understandable language, approximately at the reading level of an 11–12-year-old, without omitting important information or introducing any misinformation. Please follow these instructions when simplifying the text: Explain all medical terms and abbreviations in plain language. Briefly describe surgical procedures or pathological findings in simple terms, preferably in parentheses after the technical term. Retain all essential information, including findings, diagnoses, operations, reports, clinical course, and outcomes. Ensure that all treatment steps and medical procedures are reproduced accurately and completely. Do not shorten the content or omit any details relevant to medical treatment. Do not invent or add any information that is not explicitly stated in the original report. Write the output as a continuous narrative text (no bullet points). Omit the tooth numbering system and instead describe the teeth or region (e.g., wisdom tooth, molar, canine, front tooth; mandible/maxilla, right/left side). A coronectomy refers to the removal of the crown of a tooth while leaving the roots in place to avoid nerve injury. “8er Ost” describes the complete removal of the wisdom teeth. “Retinierte und verlagerte 8er” refers to impacted and displaced wisdom teeth. Treatment steps such as smoothing bone edges, removal of inflamed tissue, or noting a positive oroantral communication (MAV) are important and must be explicitly included in the simplified version.

Pathology report:
Rewrite the following medical report in simple and understandable language, approximately at the reading level of an 11–12-year-old, without omitting important information or introducing any misinformation. Please follow these instructions when simplifying the text: Explain all medical terms and abbreviations in plain language. Briefly describe surgical procedures or pathological terms in simple language, preferably in parentheses after the technical term. Retain all essential information, including findings, diagnoses, operations, reports, clinical course, and outcomes. Ensure that all treatment steps and medical procedures are represented accurately and completely. Do not shorten or omit any information relevant to medical treatment. Do not add or invent any content that is not explicitly stated in the original report. Write the output as a continuous narrative text (no bullet points). Omit the tooth numbering system, and instead describe the tooth or region (e.g., wisdom tooth, molar, canine, front tooth; mandible/maxilla, right/left side).

Discharge summary:
Rewrite the following medical report in simple and understandable language, without omitting important information or introducing any misinformation. Please follow these instructions when simplifying the text: Write the report at a readability level corresponding to a Flesch Reading Ease Score of 80–89 and a LIX readability index of 25–30. Explain all medical procedures, pathological terms, and abbreviations in plain language (preferably in parentheses after the technical term). Retain all essential information, including findings, diagnoses, operations, reports, clinical course, and outcomes. Ensure that all treatment steps and medical procedures are represented accurately and completely. Do not shorten or omit any information relevant to the medical treatment. Do not invent or add any content that is not explicitly mentioned in the original report. Write a continuous narrative text (no bullet points) and preserve the structure and sectioning of the original report. Omit the tooth numbering system and instead describe the teeth or region (e.g., wisdom tooth, molar, canine, front tooth; mandible/maxilla, right/left side). Explain that a neck dissection means that lymph nodes in the neck area are surgically removed to eliminate potential cancer spread. In the section “Additional Consultation”, clarify that after re-operation, an R0 status (complete tumor removal, i.e., no residual tumor) was achieved — but do not yet explain the R1 status here. In the section “Histopathological Findings”, explain that a histological R1 resection status indicates that a small part of the tumor remained and therefore required further surgical removal.

Questionnaire:

Rating questions:
The questionnaire consisted of 9 questions divided into three thematic categories: 
Comprehensibility
I found the report easy to understand. (Question number 1)
The medical terms in the radiology report were clearly explained. (Question number 2)
I was able to understand the report without additional help. (Question number 3)
Structure and Information Content
The report had a clear structure that helped me to easily understand the information. (Question number 4)
I felt that all important information was included and that the report conveyed the key points. (Question number 5)
Reading the report made me feel better informed and more involved in the treatment process. (Question number 6)
Application and interaction
The report helps me ask the right questions to my physician. (Question number 7)
After reading the report, I can discuss my diagnosis with my physician. (Question number 8)
I would like all of my medical reports to be presented in this clear and understandable way. (Question number 9)

Questions were answered on a 5-point Likert scale ranging from 1 (“do not agree at all”) to 5 (“fully agree”), with intermediate options: “rather do not agree” (2), “neither agree nor disagree” (3) and “rather agree” (4).

Comprehension questions:

MRI report
1. What is the articular disc of the temporomandibular joint (TMJ)?
The bony part of the joint
The fibrous component of the joint
The cartilage of the joint
The muscular part responsible for jaw movement

2. On which side does the disc not return to its normal position during mouth opening?
Left
Right
Neither side returns
Both sides return

3. What is a cine sequence in MRI?
An MRI scan with contrast agent
An MRI scan showing moving images
A static MRI scan
A full-body MRI scan

4. Which statement about the additional findings is correct?
The eye sockets showed pathological changes
The paranasal sinuses were not aerated
The bone marrow showed marked abnormalities
The brain showed no fluid accumulation
CT report
1. An abscess is …
A malignant tumor
Cancer
A collection of pus
A fluid-filled cyst forming around a tooth

2. Where is the abscess located?
Right side below the lower jaw
Left side below the lower jaw
Right side of the upper jaw
Left side of the upper jaw

3. Which statement is correct?
No swelling is present
Compared to the previous 3D jaw scan, bone destruction has progressed further
No contrast agent was used
There is no previous 3D scan available for comparison

4. Which statement regarding additional findings is correct?
The paranasal sinuses show abnormalities
There is no remaining tooth root in the lower jaw
The report contains no additional findings
The spine and intervertebral discs show degenerative changes

Surgical report
1. Which surgical procedure was performed?
Removal of salivary stones
Simple extraction of anterior teeth
Wisdom tooth surgery, with removal of only the crown of one tooth
Incision and drainage of an abscess

2. Where was the tooth located from which only the crown was removed?
Lower jaw, right side
Upper jaw, right side
Lower jaw, left side
Upper jaw, left side

3. Why was only the crown removed from one tooth?
It was not possible to remove the entire tooth through the surgical access
The tooth was located too close to an important nerve
To save time, tooth roots can sometimes be left in the jaw
Only the crown represents a source of inflammation and therefore a risk

4. What postoperative care instructions were given to the patient?
Keep the head warm and engage in physical activity
Take hot baths and eat normally
Blow the nose regularly and consume hard foods
Cool the head, keep it elevated, and avoid physical exertion

Pathology report
1. A follicular cyst is …
A malignant tumor
Cancer
A fluid-filled cyst that forms around a tooth
A collection of pus

2. In a biopsy report, the macroscopic description refers to …
The tissue sample taken from the patient and submitted for analysis
What is visible under the microscope
The final biopsy result
The surgical procedure itself

3. What was the overall conclusion of the pathology report?
There are indications of cancer
There are no indications of cancer
The cysts are malignant
The jawbone is severely damaged

4. In which tissue sample was no follicular cyst detected?
Tissue sample 1
Tissue sample 2
Tissue sample 3
Tissue sample 4

Discharge summary: 
1. What was not performed during surgery?
Removal of the tumor including a section of the lower jawbone
Removal of lymph nodes on both sides of the neck
Removal of the tumor including a section of the upper jawbone
Replacement of a section of the lower jawbone with a piece of leg bone

2. What is meant by a tumor board?
A group of specialists who meet to discuss the individual patient case and make treatment recommendations
A collection of all radiographic images
A compilation of all laboratory results
A clinical guideline

3. Which complication occurred on the third postoperative day?
A heart attack
An inflamed and ruptured diverticulum in the large intestine
A stroke
A wound healing disorder, which resulted in poor bone healing

4. Which statement is correct?
The tumor board advised against lymph node removal
The patient is allowed to eat normally after the operation
The patient no longer needs follow-up care
Radiation therapy was recommended


Statistical results for patient ratings across document types

MRI
CT
Surgical
Pathology
Discharge

U
p
n
U
p
n
U
p
n
U
p
n
U
p
n
1
173
< 0.01
51/45
193
< 0.01
42/51
131
< 0.01
48/47
205
< 0.01
49/45
270
< 0.01
46/39
2
254
< 0.01
51/45
205
< 0.01
42/51
108
< 0.01
48/47
251
< 0.01
49/45
156
< 0.01
46/38
3
215
< 0.01
51/45
219
< 0.01
42/51
158
< 0.01
48/47
277
< 0.01
49/44
241
< 0.01
46/38
4
264
< 0.01
51/45
414
< 0.01
42/51
309
< 0.01
48/47
324
< 0.01
49/44
297
< 0.01
46/38
5
209
< 0.01
51/45
448
< 0.01
42/51
340
< 0.01
48/47
334
< 0.01
49/44
357
< 0.01
46/38
6
237
< 0.01
51/45
329
< 0.01
42/51
212
< 0.01
48/47
238
< 0.01
49/44
306
< 0.01
46/38
7
200
< 0.01
51/45
352
< 0.01
42/51
195
< 0.01
47/47
372
< 0.01
49/44
380
< 0.01
46/38
8
248
< 0.01
51/45
355
< 0.01
42/51
206
< 0.01
47/47
429
< 0.01
49/44
339
< 0.01
46/38
9
260
< 0.01
51/45
311
< 0.01
42/51
143
< 0.01
48/47
255
< 0.01
49/44
254
< 0.01
46/38
Table 2: Results of the Mann–Whitney U-tests comparing simplified and original versions of five medical document types (MRI report, CT report, surgical report, pathology report, and discharge summary) across nine questionnaire items. Each item was rated by patients on a five-point Likert scale ranging from 1 (“do not agree at all”) to 5 (“fully agree). For each comparison, the table lists the U-value, the corresponding p-value, and the group sample sizes (n). Significant p-values (< 0.05) indicate statistically higher ratings for the simplified versions.
